# Effect of adding *Scutellaria baicalensis* Georgi to feed on the microbial diversity in cattle feces

**DOI:** 10.3389/fmicb.2025.1641802

**Published:** 2025-08-22

**Authors:** Junli Zhang, Xue Bai, Fen Li, Rina Na, Yun Ma, Xiaojun Liang

**Affiliations:** ^1^Institute of Animal Science, Ningxia Academy of Agriculture and Forestry, Yinchuan, Ningxia, China; ^2^Key Laboratory of Ruminant Molecular and Cellular Breeding, College of Animal Science and Technology, Ningxia University, Yinchuan, China; ^3^School of Food Science and Engineering, Ningxia University, Yinchuan, Ningxia, China

**Keywords:** beef cattle, *Scutellaria baicalensis* Georgi, production performance, biochemical indicators, fecal microbes

## Abstract

**Introduction:**

Inflammation and oxidative stress can seriously endanger the health and growth of beef cattle. *Scutellaria baicalensis* Georgi (SB) has significant anti-inflammatory and antioxidative effects. However, studies on the application of SB stems and leaves as roughage in animal husbandry are limited. Therefore, in this study, we investigated the effects of adding different proportions of SB stems and leaves to roughage on the microbial diversity of bovine feces.

**Methods:**

In total, 20 cattles (Simmental xlocal yellow cattle) were randomly divided into four groups: CON, G1, G2, and G3 (with 3%, 6%, and 9% SB stems and leaves added to the roughage, respectively). All cattles were exposed to pre-experimental conditions for 15 days and experimental conditions for 90 days. Growth performance indicators were measured on the 0th, 15th, 45th, and 90th days of the formal experiment. Blood was collected for measuring biochemical performance indices. Fresh fecal samples were collected on the 90th day for 16S sequencing to study the microbial diversity of cow feces.

**Results:**

The results showed that during the experiment, the F/G of the G1, G2, and G3 groups was significantly lower than that of the CON group (*P* < 0.05). The IL-10 content in the serum of the G3 group was significantly greater than that in the CON group (*P* < 0.05). The 16S rRNA results revealed significant differences in the composition of the bacterial community between the SB groups and the CON group. At the phylum level, adding the stems and leaves of SB significantly decreased the Firmicutes/Bacteroidetes ratio, and the relative abundances of Spirochaetes, Verrucomicrobia, and Proteobacteria decreased. At the genus level, *Anaerovibrio, Epulopiscium, Akkermansia, Bacteroides*, YRC22, *Paludibacter*, 5-7N15, *Butyrivibrio, Prevotella*, and *Anaeroplasma* were identified as markers of intergroup differences. These intergroup differential microbiota were associated with pathways such as amino acid biosynthesis, fatty acid and lipid biosynthesis, carbohydrate biosynthesis, carbohydrate degradation, nucleoside and nucleotide degradation, fermentation, glycolysis, the TCA cycle, glycan biosynthesis, and glycan degradation.

**Discussion:**

Our findings revealed that adding the stems and leaves of SB to feed can promote body growth and antioxidant capacity and increase the diversity of the gut microbiota. This new feeding strategy can improve the efficiency of cattle farming.

## Introduction

With the rapid development of the Chinese traditional herbal medicine (CTHM) industry, the scope and scale of Chinese herbal medicine cultivation have been continuously expanding. Chinese herbal medicine can be stored in large quantities. It has the advantages of low production costs, good prevention and control effects, and negligible toxic side effects. CTHM has been used as a new type of feed in practical production. Many studies have investigated the application of Chinese herbal medicines as livestock and poultry feed. Wang et al. reported that adding Guizhi Li-Zhong Tang (GLZ) to piglet feed can prevent pneumonia in weaned piglets (Wang C. H. et al., 2021). Chen et al. reported that adding fermented Chinese herbal medicine to feed can promote growth performance, improve intestinal health, and regulate bacterial flora in weaned piglets (Chen G. et al., 2023). Among them, *Scutellaria baicalensis* Georgi (SB) is an important Chinese herbal medicine widely cultivated in northwestern China (Song et al., 2020). In the Ningxia region of China, the selling price of the stems and leaves of SB is 1 RMB/kg. In comparison, the selling price of local corn is 2 RMB/kg. Thus, the cost of the stems and leaves of SB is only half of that of corn. Additionally, SB contains various chemical components, such as flavonoids, diterpenes, polyphenols, amino acids, volatile oils, sterols, and benzoic acid (Dinda et al., 2017; Yan et al., 2017). These are the main compounds in SB that exert clinical effects. The stems and leaves of SB contain nutrients such as flavonoids, saponins, sugars, amino acids, and various trace elements, which have pharmacological effects such as clearing heat, drying dampness, purging fire (Bai et al., 2020), detoxifying (Zhou et al., 2021), anti-inflammatory (Yu et al., 2019), antitumor (Ke et al., 2019), antioxidant (Zhao et al., 2019) and immune regulation (Shin et al., 2014). Concerning human diseases, studies have shown that Huangcen and its extract can effectively treat type II diabetes (Yingrui et al., 2022), lung cancer (Xiang et al., 2022), autoimmune diseases (Wang J. et al., 2022), ovarian cancer (Cai et al., 2023), breast cancer (Yu et al., 2023), hepatitis, and colitis (Jang et al., 2023). In animal experiments, Wang et al. reported that baicalin, the main bioactive extract of SB, can protect against LPS-induced damage to the blood-brain barrier and activate the Nrf2-mediated antioxidant stress pathway (Wang X. et al., 2021). Qian et al. reported that SB can increase neurogenesis and improve memory induced by the PI3K-AKT-CREB signaling pathway in mice (Liu Q. Q. et al., 2022). Sun et al. reported that the extract of polysaccharide (SPS) from SB improves the immunity and antioxidant capacity of giant freshwater prawns (Sun et al., 2023). Zhang et al. reported that adding SB to piglet feed can alter the gut microbiota composition of piglets, which can increase the weaning weight and promote piglet health (Zhang F. et al., 2022). However, few studies have investigated the addition of the stems and leaves of SB to ruminant silage feed. Zong et al. reported that the intestinal microbiota of pigs of different breeds has common and specific regulatory effects on host genes (Huang et al., 2024). This finding showed that research at the level of host microorganisms and genes holds a crucial position. With the advancement of microbial sequencing technology, in this study, we aimed to assess the effect of adding different proportions of SB to feed on the microbial diversity of cow feces. Our findings provided a theoretical basis for adding traditional Chinese medicine formulations to cattle feed.

The gut microbiota, found in the gastrointestinal tract of animals, greatly influences the health of the host. The gut microbiota has many positive effects on the host, including the maturation, metabolism, and physiological function of the immune system, as well as the protection of pathogens (Barko et al., 2017). Sun et al. showed that increasing quercetin in the basal diet can alleviate the inflammatory response of broilers and regulate the jejunal microbiota (Sun et al., 2022). (Dou et al. 2023) suggested that supplementation with *Clostridium butyricum* in the diet can alter the gut microbiota by regulating the intestinal muscle axis and affecting the skeletal muscle development and meat quality of lambs. Zhang et al. reported that adding different proportions of *Cistanche deserticola* to feed can alter the rumen microbial structure of sheep. Adding *C. deserticola* can improve the rumen fermentation environment and regulate the rumen microbial community structure (Zhang X. et al., 2022). Winders et al. reported that feeding hempseed cake increased the diversity of the rumen and intestinal microbiota in heifers. (Zeng et al. 2024) found that the adjuvant use of *Lactobacillus paracasei* GY-1 with colchicine can alleviate intestinal toxicity and increase the efficacy of gout treatment. Different ingredients in feed can alter the gut microbiota structure of livestock, thereby affecting their growth, development, and health (Winders et al., 2023). Although previous studies have found that SB is beneficial for human and animal health, there are few reports on the addition of SB to cattle feed and the role of gut microbiota in it. Therefore, we studied the changes in cattle health status after adding SB by measuring the production performance and serum biochemical indicators of cattle, and clarified the differences in fecal microbiota that led to this result through 16S rRNA gene sequencing, providing a theoretical basis for promoting cattle health using locally inexpensive Chinese herbs.

## Materials and methods

### Animals and feeding strategy

All protocols for animal handling and sampling were approved by the Animal Care and Use Committee of Ningxia Academy of Agriculture and Forestry (2022-13), and the study was conducted following the approved protocol. The 20 cattles (Simmental × local yellow cattle) weighing 385.67 ± 39.21 kg were randomly divided into four groups, with 5 cattles in each group (One cow in the CON group died due to its own health problems, which does not affect the comparability between groups and the reliability of the conclusions). The experimental diet was prepared as per the NY/T 815–2004 (Beef Cattle Feeding Standards, 2004) standard. In the CON group, SB was not added, whereas in the experimental group, 3%, 6%, and 9% SB were added. The main active ingredient of the stems and leaves of SB used in this study was baicalin, and its content was 0.046% (Table 1). The specific dietary composition and nutritional levels are shown in Table 2. Each cow was supplemented with 1 kg of a certain commodity beef concentrate supplemented with pellet feed every day; its nutritional composition is presented in Table 3. All experimental beef cattle were subjected to a pre-trial period of 15 days and were fed for 90 days.

**Table 1:** Nutritional components of the stems and leaves of *Scutellaria baicalensis* Georgi.

**CP (%)**	**NDF (%)**	**ADF (%)**	**EE (%)**	**Crude fiber (%)**	**ASH (%)**
7.63	59.17	47.89	0.57	0.93	0.56

NDF, Neutral detergent fiber; ADF, Acid detergent fiber; EE, Crude fat.

**Table 2:** Composition and nutrient levels of the total mixed rations.

**Items**	**CON group**	***Scutellaria baicalensis*** **Georgi addition group (%)**		
		**G1**	**G2**	**G3**
**Ingredient**				
Straw	6.00	5.00	5.00	4.00
Reed grass	7.00	5.00	3.00	3.00
Corn cob	15.00	15.00	15.00	15.00
Corn silage	24.00	24.00	24.00	24.00
Wolfberry leaves and rake	1.20	1.20	1.20	1.20
*Scutellaria baicalensis* Georgi	0.00	3.00	6.00	9.00
Wheat straw	6.00	6.00	5.00	3.00
Corn	27.00	27.00	27.00	27.00
Flax meal	6.00	6.00	6.00	6.00
Wheat bran	6.00	6.00	6.00	6.00
NaHCO3	0.50	0.50	0.50	0.50
NaCl	0.40	0.40	0.40	0.40
Premix^a^	0.90	0.90	0.90	0.90
Total	100.00	100.00	100.00	100.00
**Nutrient levels**				
CF	18.6	19.34	20.08	20.82
CP	7.42	7.43	7.43	7.44
Ca	0.48	0.47	0.46	0.45
P	0.30	0.29	0.29	0.28
NE_mf_ (MJ/kg)	5.81	5.75	5.70	5.64

The premix provided the following per kg of finished product: VA 40 000–330 000 IU, VD_3_ 10 000–100 000 IU, VE 600 000 IU, Fe 60–1 600 mg, Zn 60–400 mg, Cu 10–500 mg, and Mg 40–500 mg. The same as below. CF, Crude fiber; CP, Crude protein; Ca, Calcium; P, Phosphorus. NE_mf_ was a calculated value, whereas the other nutrient levels were measured values. In the same row, values with different small letter superscripts are significantly different (*P* < 0.05), whereas values with the same or no letter superscripts are not significantly different (*P* > 0.05).

**Table 3:** Nutrient levels of the beef concentrate supplemented with pellet feed.

**CP (%)**	**NDF (%)**	**ADF (%)**	**EE (%)**	**Ca (%)**	***P* (%)**	**NE_mf_/(MJ/kg)**
18.52	8.51	23.22	5.36	0.93	0.56	6.74

NDF, neutral detergent fiber; ADF, acid detergent fiber; EE, crude fat.

### Determination of production performance

The feeding amount was recorded daily, the feeding trough was cleaned and swept before feeding the following day, the remaining amount of feed from the previous day was weighed, and the feed intake was calculated. Body weight was measured on days 0, 15, 45, and 90 during the formal feeding period. The average daily gain (ADG), dry matter intake (DMI), and feed-to-weight ratio (F/G) were calculated.

### Sample collection and processing

We collected 10 mL of carotid artery blood from each beef cow on the morning of the 90th day of the formal trial. The mixture was left undisturbed at room temperature for 30 min, the blood sample was allowed to coagulate, the bleeding serum was precipitated in the blood collection vessel, and the mixture was centrifuged at 3,000 r/min for 15 min. A pipette was used to dispense the upper layer of transparent serum, which was frozen at −20 °C for storage. Fresh feces were collected from beef cattle on the 90th day of the formal trial. First, the beef cattle were fixed with neck clips, and fresh feces were collected from the rectum at 7:00, 12:00, 17:00, and 22:00 h. The feces were stored in liquid nitrogen and frozen for microbial diversity analysis (16S V3-V4).

### Detection of serum biochemical indicators

The biochemical indicators of bovine serum were measured using a Mindray BS-420 fully automatic biochemical analyzer and a Huawei Langde DR-200BS instrument with the assistance of the Beijing Huaying Biotechnology Research Institute. GLU (glucose, HY-50063), TP (total protein, HY-50067), ALB (albumin, HY-50068), UREA (urea nitrogen, HY-N0015), CREA (creatinine, HY-N0017), CK (creatine kinase, HY-50056), CHE (serum cholinesterase, HY-N0046), IgA (immunoglobulin A, bs-0360Rs), IgG (immunoglobulin G, bs-0297R), IgM (immunoglobulin M, bs-0345R), T-AOC (total antioxidant capacity, HY-60021), SOD (superoxide dismutase, HY-M0001), GSH-PX (glutathione peroxidase, HY-M0004), IL-2 (interleukin-2, bs-1191P), IL-6 (interleukin-6, bs-0379P), IL-10 (interleukin-10, bs-0698P), and TNF-ɑ (tumor necrosis factor- ɑ, bs-0078P) in the serum were detected using a reagent kit manufactured by Beijing Huaying Biotechnology Research Institute.

### 16S rRNA sequencing

The total genomic DNA of the sample was extracted using the MP Biomedicals Kit (America, MP Biomedicals), and the concentration and purity of DNA were measured using a 1% agarose gel. The concentration of each DNA sample was diluted to 1 ng/μL with sterile water. The 16S rRNA genes in different regions were amplified using specific primers with a bar code (515F/806R). Then, 15 μL of Phusion was added to the PCR mixture for high-fidelity PCR with the main mixture (New England Biological Laboratory) using 2 μM forward and reverse primers and about 10 ng of template DNA. The thermal cycle included initial denaturation at 98 °C for 1 min, denaturation at 98 °C for 30 cycles for 10 s, annealing at 50 °C for 30 s, extension at 72 °C for 30 s, and a final hold at 72 °C for 5 min. The same volume of 1 × loading buffer (including SYBR Green) was mixed with the PCR product and detected by 2% agarose gel electrophoresis.

The PCR products were mixed at an equal density ratio. The Qiagen Gel Extraction Kit (German Qiagen) was used to purify the mixed PCR products. TruSeq was recommended by the manufacturer of the DNA-free PCR sample preparation kit (Illumina, United States) to generate the sequencing library and add the index code. The quality of the library was evaluated using a Qubit@ 2.0 fluorometer (Thermo Scientific) and an Agilent Biological Analyzer 2100 system. Finally, the library was sequenced on the Illumina NovaSeq platform, and 250-bp paired-end read codes were generated.

Each sample dataset was separated from the offline data according to the bar code and PCR-amplified primer sequences. The bar code and primer sequences were intercepted with FLASH (V1.2.7, http://ccb.jhu.edu/software/FLASH/). The reads of each sample were spliced, and the spliced sequences were the original data tags (raw tags). We referred to the QIIME (V1.9.1, http://qiime.org/scripts/split_libraries_fastq.html) process to obtain a high-quality tag sequence. These tag sequences were passed (https://github.com/torognes/vsearch/). The annotation databases of the species were compared to detect chimeric sequences. Adopting the QIIME2 classification sklarn algorithm (https://github.com/QIIME2/q2-feature-classifier) for each ASVs feature sequence or representative sequence of each OTU, use default parameters in QIIME2 software and perform species annotation using a pre trained Naive Bayes classifier. Classification allocation is based on the Greengenes database (Rlease 13.8, https://mothur.org/wiki/greengenes-formatted_databases/). Shannon index and principal coordinate analysis (PCoA) based on Bray Curtis dissimilarity were calculated using R package voxels. Statistical analysis of alpha diversity and beta diversity using Wilcoxon rank and BH multiple comparisons (*p*-value < 0.05 for FDR correction is considered significant).

Using the UPARSE algorithm (UPARSE v7.0.1001, http://www.drive5.com/uparse/), all effective tags of the samples were clustered. By default, the sequences were clustered into operational taxonomic units (OTUs) with 97% identity. Moreover, the sequence with the highest frequency of OTUs was selected as the representative sequence of OTUs. The Music (version 3.8.31, http://www.drive5.com/muscle/) software was used to perform fast multisequence alignment to determine the phylogenetic relationships of representative OTU sequences.

### Statistical analysis

All statistical analyses were conducted using the SPSS 25.0 software (IBM, Chicago, Illinois, United States). The differences among the four groups were determined by conducting two-way ANOVA. To conduct the similarity analysis of multivariate data (Anosim), the R software (http://www.r-project.org/) was used to compare the bacterial community structure. All results were considered to be statistically significant at *P* < 0.05. In addition, the topological characteristics of the co-occurrence network constructed using the subgraph function in the R package graph; Use the uclust function in the Stat package of R language, default to using UPGMA algorithm for the Bray Curtis distance matrix, and use the R script ggtree package for visualization.

## Results

### The effect of adding SB on the production performance of cattle

By measuring the production performance of beef cattle during the feeding period, the body weights of beef cattle in all four experimental groups increased during the feeding period. At the beginning of the formal experiment, no significant difference was found in the initial body weight (IBW) of the Simmental crossbred cattle in each group (*P* > 0.05). After the experiment ended, compared to those in the CON group, the final body mass (FBM) and ADG of Simmental crossbred cattle in Group G2 were significantly greater than those in the other groups (*P* < 0.05). The F/G ratios of Groups G1, G2, and G3 were significantly lower than those of the CON group (*P* < 0.05) (Table 4). As shown in Table 5, compared to that of the CON group, the weight gain income of the G1, G2, and G3 groups increased by 424.8, 1,233, and 811.8 yuan, respectively.

**Table 4:** Effects of SB feeding on production performance of Simmental hybrid cattle.

**Items**	**CON**	**SB addition group/%**		
		**G1**	**G2**	**G3**
Initial body weight (IBW, kg)	373.75 ± 48.68	370.00 ± 42.59	377.64 ± 62.72	366.08 ± 44.31
Final body weight (FBW, kg)	431.90 ± 40.17^b^	434.81 ± 50.39^b^	469.02 ± 85.05^a^	445.28 ± 56.72^b^
Average daily weight gain (ADG, kg/d)	0.97 ± 0.19^b^	1.08 ± 0.51^b^	1.52 ± 0.34^a^	1.32 ± 0.20^a^
Dry matter intake (DMI, kg/d)	9.78 ± 1.45	8.49 ± 1.12	9.28 ± 1.53	8.50 ± 1.29
feed-gain ratio (F/G)	21.72 ± 1.41^a^	16.09 ± 3.14^b^	14.69 ± 2.40^b^	13.85 ± 1.74^b^

In the same row, values with different small letter superscripts are significantly different (P < 0.05), whereas values with the same or no letter superscripts are not significantly different (P > 0.05).

**Table 5:** Economic benefits of adding the stems and leaves of SB to the diet of beef cattle.

**Groups**	**Fattening day (d)**	**Daily feeding cost (yuan/head)**	**Average daily weight gain (kg/head)**	**Daily gain income (yuan/head)**	**Daily profit (yuan/head)**	**Total profit (yuan/head)**	**Beef cattle live weight price**	**Profit/yuan**
CON	60	15.71	0.97	34.92	19.21	1,152.6	36	0
G1	60	12.59	1.08	38.88	26.29	1,577.4	36	424.8
G2	60	14.96	1.52	54.72	39.76	2,385.6	36	1,233
G3	60	14.78	1.32	47.52	32.74	1,964.4	36	811.8

### The effects of adding SB on the biochemical indicators of cattle serum

The analysis of serum biochemical indicators (Table 6) revealed that at the end of the formal experiment, the IL-10 content in the serum of the G3 group was significantly lower than that in the CON group *(P* < 0.05). No significant changes were found in various biochemical indicators in the CON, G1, G2, and G3 groups (*P* > 0.05). However, we identified several trends. Compared to those in the CON group, the serum TP, GLB, CREA, CHE, MDA, IL-2, IL-6, and TNF-α levels in the G1, G2, and G3 groups were lower, whereas the GLU, IgA, IgG, IgM, SOD, T-AOC, and IL-10 levels were higher.

**Table 6:** Effects of adding SB on the biochemical indicators of cattle serum.

**Items**	**CON**	**SB addition group/%**		
		**G1**	**G2**	**G3**
Total protein (TP, g/L)	64.44 ± 9.37	58.30 ± 8.00	58.74 ± 13.03	57.65 ± 8.37
Albumin (ALB, g/L)	26.89 ± 4.28	24.94 ± 4.76	25.25 ± 6.30	25.31 ± 3.98
globulin (GLB, g/L)	37.55 ± 6.32	33.36 ± 3.47	33.50 ± 6.86	32.34 ± 4.52
glucose (GLU, mmol/L)	0.75 ± 0.23	0.90 ± 0.43	1.08 ± 0.77	1.15 ± 0.29
UREA (mmol/L)	1.23 ± 0.41	1.09 ± 0.47	1.38 ± 0.50	1.34 ± 0.43
creatinine (CREA, μmol/L)	50.34 ± 11.89	48.25 ± 9.11	47.31 ± 20.63	49.58 ± 20.63
cholinesterase (CHE, U/L)	526.93 ± 78.13	476.59 ± 25.01	470.12 ± 15.01	488.61 ± 19.51
Immunoglobulin A (IgA, g/L)	0.82 ± 0.05	0.84 ± 0.10	0.96 ± 0.12	0.94 ± 0.09
Immunoglobulin G (IgG, g/L)	10.73 ± 1.99	11.30 ± 1.09	11.09 ± 2.54	11.51 ± 1.18
Immunoglobulin M (IgM, g/L)	2.62 ± 0.65	2.41 ± 0.36	2.51 ± 0.61	2.27 ± 0.39
Superoxide Dismutase (SOD, U/mL)	39.54 ± 2.41	40.53 ± 3.86	42.84 ± 6.36	46.00 ± 10.52
Malondialdehyde (MDA, nmol/mL)	3.71 ± 0.36	3.32 ± 0.55	3.25 ± 0.65	3.16 ± 0.35
glutathione peroxidase (GSH-PX, U/mL)	286.77 ± 66.01	275.09 ± 54.91	291.73 ± 99.36	295.70 ± 14.28
Total antioxidant capacity (TAOC, U/mL)	10.24 ± 0.96	13.93 ± 2.34	14.83 ± 4.02	15.06 ± 2.41
Interleukin-2 (IL-2, pg/mL)	329.96 ± 54.13	307.96 ± 11.20	303.64 ± 21.66	273.21 ± 24.80
Interleukin-6 (IL-6, pg/mL)	151.93 ± 27.37	139.25 ± 10.54	140.28 ± 12.44	134.66 ± 10.86
tumor necrosis factor-α (TNF-α, pg/mL)	76.98 ± 17.86	68.08 ± 15.16	69.24 ± 10.36	60.23 ± 7.90
Interleukin-10 (IL-10, pg/mL)	18.80 ± 1.54^a^	20.21 ± 1.55^ab^	22.25 ± 3.22^ab^	24.31 ± 3.36^b^

In the same row, values with different small letter superscripts are significantly different (P < 0.05), whereas values with the same or no letter superscripts are not significantly different (P > 0.05).

### Sequence processing

Fresh fecal samples were obtained from the cattle in the CON, G1, G2, and G3 groups after 105 days of treatment to investigate the effects of adding 3%, 6%, or 9% SB on the intestinal microbiota composition of the cattle. A total of 113,457 labels were matched with 19 samples, with an effective quality control rate of 89.30% and a high quality control rate of 85.96% (Table 7). A taxonomic annotation was conducted on the microorganisms according to the comparative database, and the results are presented in Table 8. The results of the taxonomic annotation revealed that the OTUs annotated to the family level were the most abundant among the four groups, followed by those annotated to the order and genus levels.

**Table 7:** Data preprocessing, statistics, and quality control.

**Group**	**Sample**	**Input**	**Raw PE**	**Effective (%)**	**High-quality sequence**	**Rate (%)**
CON	1	5,787	4,985	86.14	4,740	81.91
	2	5,138	4,496	87.50	4,262	82.95
	3	4,555	4,345	95.39	4,295	94.29
	4	5,665	4,869	85.95	4,637	81.85
G1	1	5,683	4,828	84.96	4,584	80.66
	2	4,858	4,563	93.93	4,467	91.95
	3	7,721	6,834	88.51	6,581	85.24
	4	6,321	5,547	87.76	5,269	83.36
	5	5,476	5,266	96.17	5,192	94.81
G2	1	6,677	5,890	88.21	5,601	83.88
	2	6,127	5,426	88.56	5,239	85.51
	3	5,496	5,163	93.94	5,074	92.32
	4	5,869	5,114	87.14	4,830	82.30
	5	5,210	4,628	88.83	4,453	85.47
G3	1	6,032	5,466	90.62	5,289	87.68
	2	6,031	5,395	89.45	5,184	85.96
	3	9,020	7,760	86.03	7,495	83.09
	4	5,209	4,965	95.32	4,855	93.20
	5	6,582	5,779	87.80	5,476	83.20
	Total	113,457	101,319	89.30	97,523	85.96

**Table 8:** Annotations of species taxonomy.

**Group**	**Sample**	**Domain**	**Phylum**	**Class**	**Order**	**Family**	**Genus**	**Species**	**Unclassified**
CON	1	10	2	2	308	552	144	10	0
	2	6	0	1	278	526	128	9	0
	3	8	0	2	283	643	137	10	0
	4	11	1	2	300	546	155	7	0
G1	1	17	0	3	306	592	141	4	0
	2	4	0	1	282	496	137	8	0
	3	10	0	0	312	542	153	10	0
	4	12	1	3	320	560	150	8	0
	5	2	3	0	243	523	135	6	0
G2	1	10	1	1	307	557	150	6	0
	2	12	0	2	296	542	131	5	0
	3	5	0	0	253	501	121	5	0
	4	16	0	1	363	645	152	7	0
	5	7	1	2	301	549	144	8	0
G3	1	5	2	2	285	525	128	6	0
	2	5	0	1	206	392	123	5	0
	3	9	1	0	275	476	123	8	0
	4	6	0	0	260	507	122	5	0
	5	7	0	2	314	625	149	8	0

### Alpha diversity analysis

**Figure 1:**
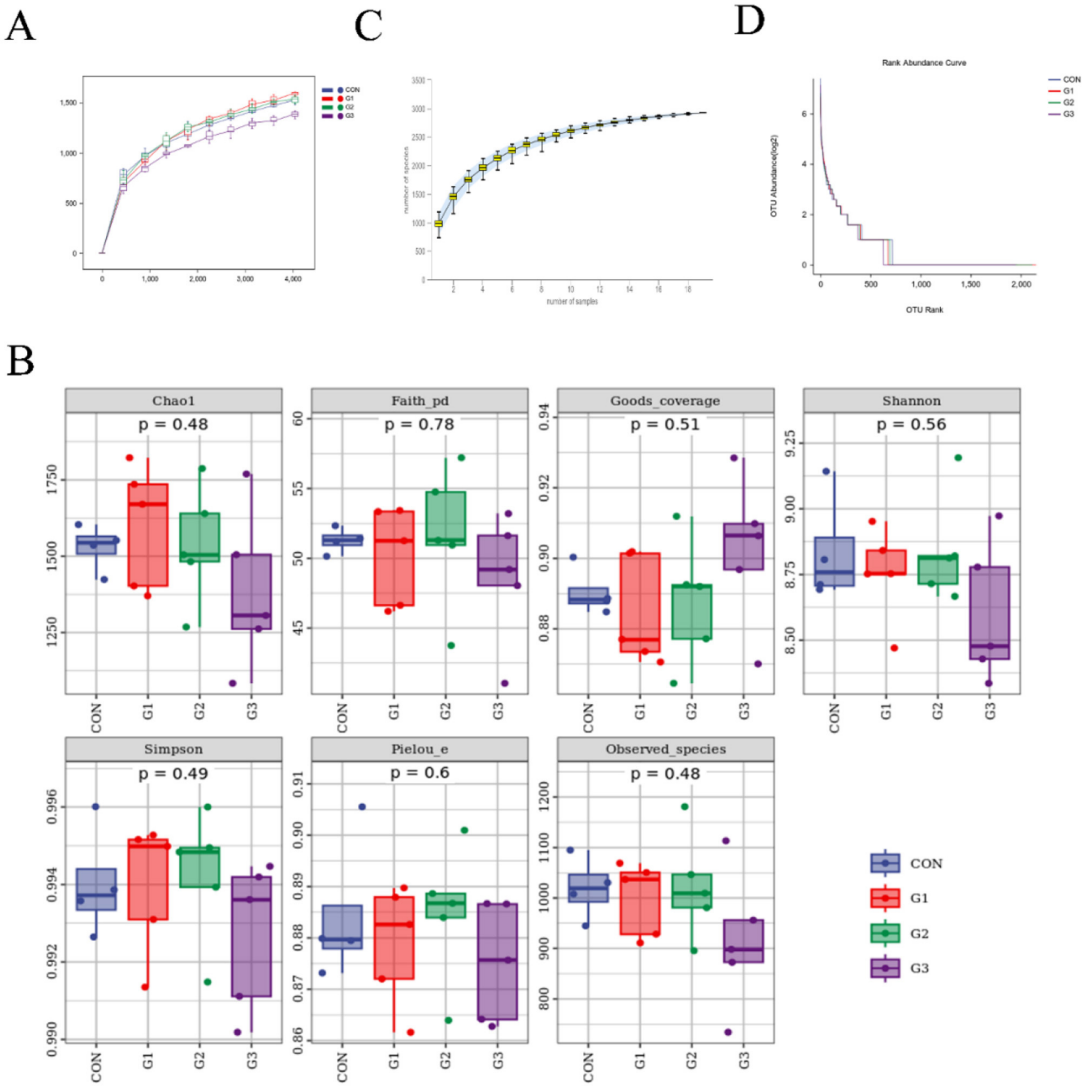
Alpha diversity analysis. **(A)** Rarefaction curves (the mean curves for the samples/group). **(B)** Box plots of the Chao, Faith, Good's coverage, Shannon, Simpson, Pielou, and observed species indices. **(C)** Species accumulation curve. **(D)** Abundance level curve.

The rarefaction curve (average curve for each group) indicated that most microbial diversity was fully recorded (Figure 1A). According to the results of the Wilcoxon rank-sum test, no significant differences were found in Chao, Faith, Goods coverage, Shannon, Simpson, Pielou or observed species (Figure 1B) among the G1, G2, G3, and CON groups (*P* > 0.05). The species accumulation curve (Figure 1C) showed rapid and stable growth, indicating high species richness, which finally reached a stable state. The rank abundance curve (Figure 1D) revealed that the species were abundant and evenly distributed.

### Beta diversity analysis

**Figure 2:**
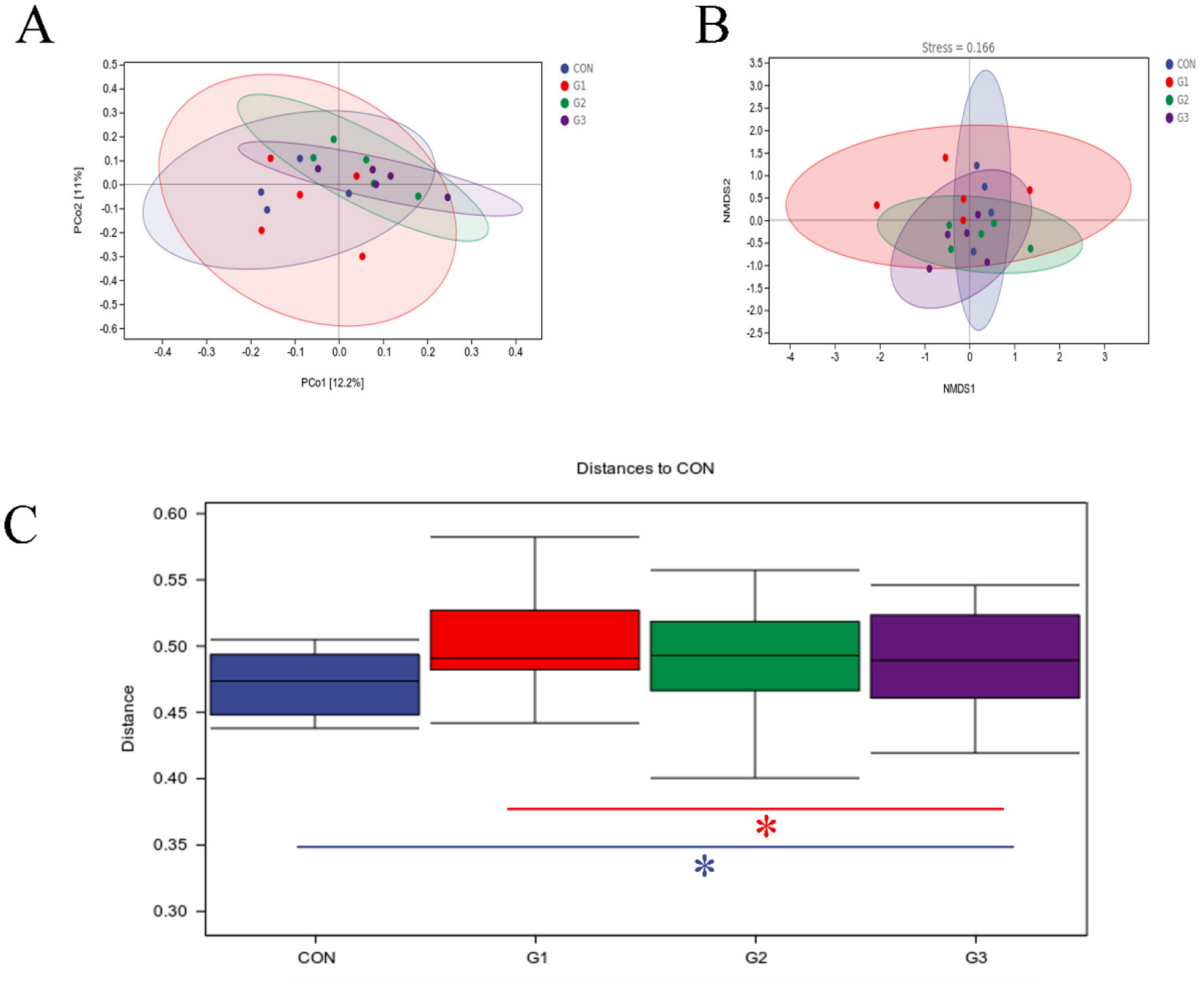
Beta diversity analysis. **(A)** The diagram illustrates PCoA. **(B)** Nonmetric multidimensional scaling (NMDS) ordination plots of the fecal bacterial communities in the CON, G1, G2, and G3 groups based on the Bray-Curtis distance metric. **(C)** The box plot shows intergroup difference analysis.

After adding SB, there were significant differences between G1, G2, G3, and CON groups. The ranking maps of PCoA (Figure 2A) and NMDS (Figure 2B) based on Bray Curtis distance measurement show significant differences among CON, G1, G2, and G3 fecal microbiota groups. The inter group differences based on ANOSIM are shown in Figure 2C. There were significant differences (*P* < 0.05) between the CON group and the G3 group, as well as between the G1 group and the G3 group.

### Phylum-level microbiota expression

**Figure 3:**
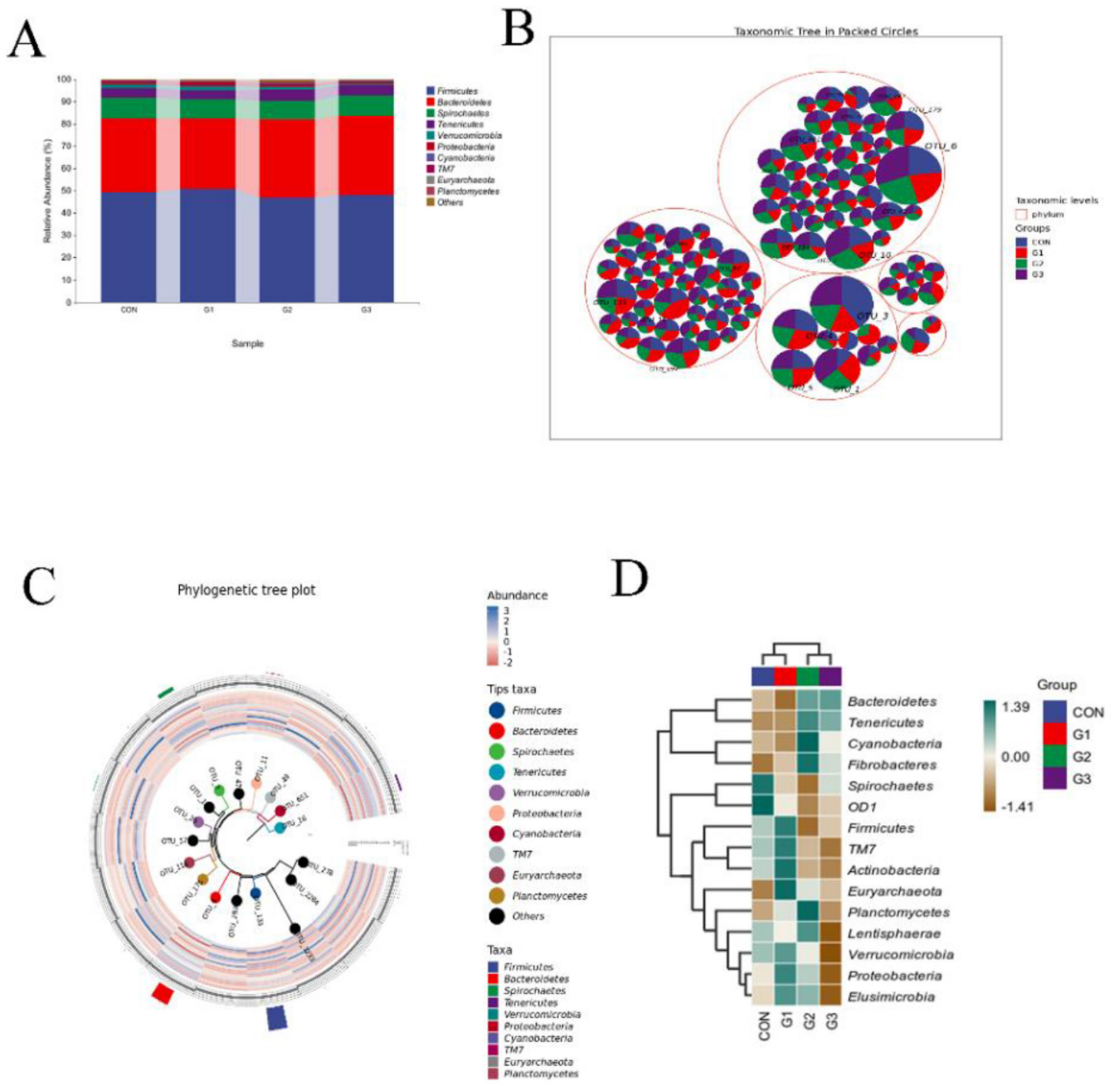
Phylum-level microbiota expression. **(A)** Composition diagram of phylum-level taxonomy. **(B)**. Taxonomic hierarchy for the phylum with the highest abundance of 20 OTUs. **(C)** Phylum-level phylogenetic tree maps. **(D)** Species composition heatmaps at the phylum level.

An analysis of the classification components with relative abundances >1% revealed that the bacterial population was affected by the addition of the stems and leaves of SB (Figure 3A). Firmicutes were the most abundant phylum in the CON group, with an average abundance of 49.47%, followed by Bacteriodota (32.81%). In the G1 group, Firmicutes (50.85%) also had an absolute advantage, followed by Bacteriodota (31.47%), Spirochaetes (8.57%), Tenericutes (4.08%), Verrucomicrobia (1.88%), and Proteobacteria (1,39%). In the G2 group, Firmicutes (46.62%) also had an absolute advantage, followed by Bacteriodota (35.57%), Spirochaetes (8.09%), Tenericutes (4.92%), Verrucomicrobia (1.43%), and Proteobacteria (1.23%). In the G3 group, Firmicutes (48.06%) also had an absolute advantage, followed by Bacteriodota (35.67%), Spirochaetes (8.90%), Tenericutes (4.76%), Verrucomicrobia (0.62%) and Proteobacteria (0.84%). We mapped the taxonomic hierarchy (Figure 3B) for the phylum with the highest abundance of 20 OTUs. We also generated phylogenetic tree maps (Figure 3C) and species composition heatmaps (Figure 3D) at the phylum level.

### Genus-level microbiota expression

**Figure 4:**
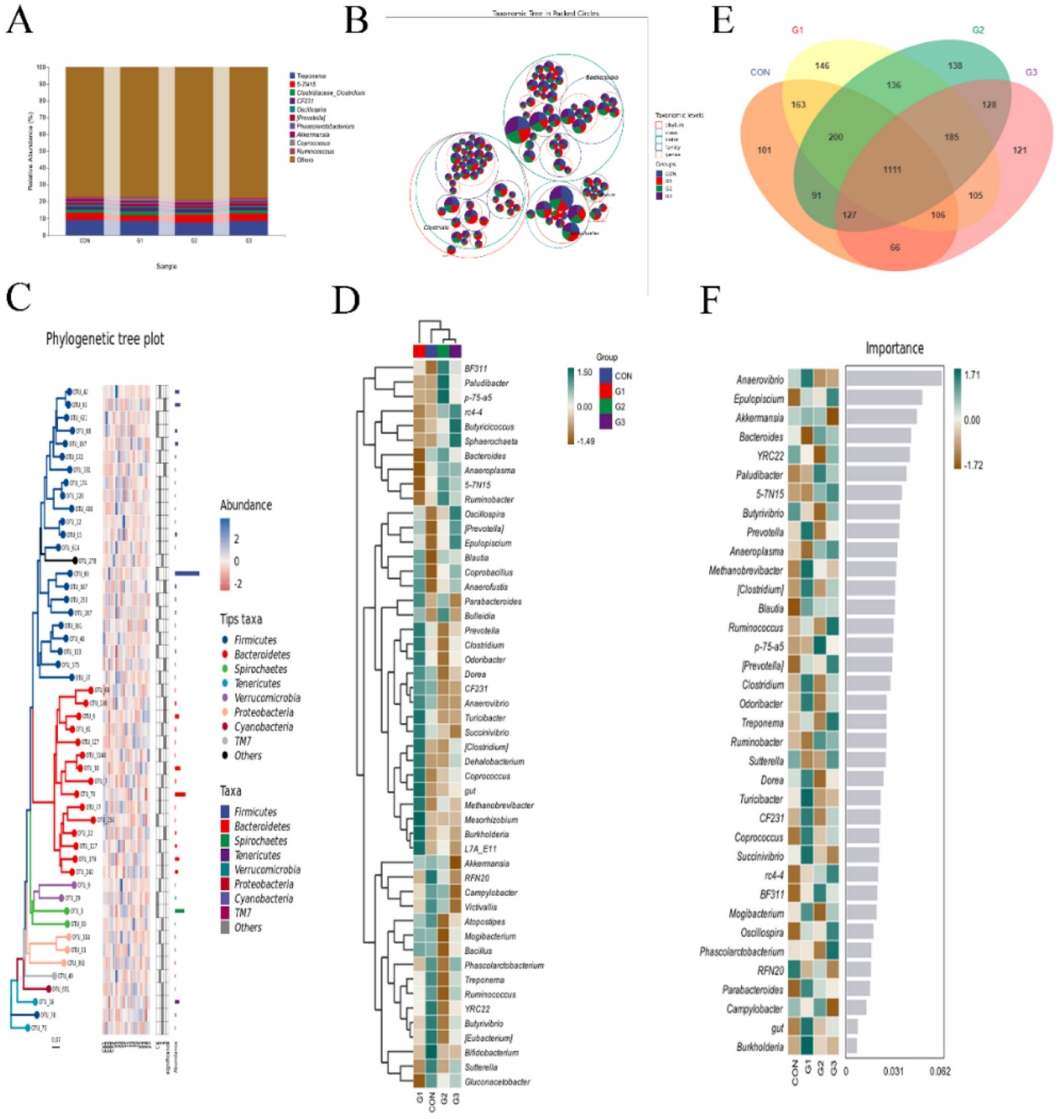
Genus-level microbiota expression. **(A)** Composition diagram of the genus-level taxonomy. **(B)** Taxonomic hierarchy for the genus with the highest abundance of 50 OTUs. **(C)** Genus-level phylogenetic tree maps. **(D)** Species composition heatmaps at the genus level. **(E)** Venn diagram of microorganisms in each group at the genus level. **(F)** Genus-level random forest analysis.

To investigate the evolutionary relationships of species at the genus level, sample sequences from the top 100 genera were acquired through multiple sequence alignment (Figure 4A). A total of 20% can be annotated to the genus level. *Treponema* was the most abundant taxon in the CON group, with an average abundance of 9.19%, followed by *5-7N15* (4.13%). In the G1 group, *Treponema* was the most abundant (8.42%), followed by *5-7N15* (3.46%), *Clostridiaceae_Clostridium* (2.31%), and *CF231* (1.9%). In the G2 group, *Treponema* was the most abundant (7.59%), followed by *5-7N15* (4.62%), *Clostridiaceae_Clostridium* (1.30%), and *CF231* (1.58%). In the G3 group, *Treponema* was the most abundant (8.36%), followed by *5-7N15* (4.34%), *Clostridiaceae_Clostridium* (1.82%), and *CF231* (1.62%). We mapped the taxonomic hierarchy (Figure 4B) for the genus with the highest abundance of 50 OTUs. We also generated phylogenetic tree maps (Figure 4C) and species composition heatmaps (Figure 4D) at the genus level. Venn diagrams and species composition heatmaps were made to further compare the differences in species composition between samples. The Venn diagram is shown in Figure 4E, with 1111 identical OTUs in the four groups. There were 101, 146, 138, and 121 OTUs in the CON, G1, G2, and G3 groups, respectively. A heatmap of the top 50 genera with average abundances is shown in Figure 4F. Among them are *Anaerovibrio, Epulopiscium, Akkermansia, Bacteroides, YRC22, Paludibacter, 5-7N15, Butyrivibrio, Prevotella*, and *Anaeroplasma*. These microorganisms serve as markers of intergroup differences.

### Enrichment of differential microbiota pathways

**Figure 5:**
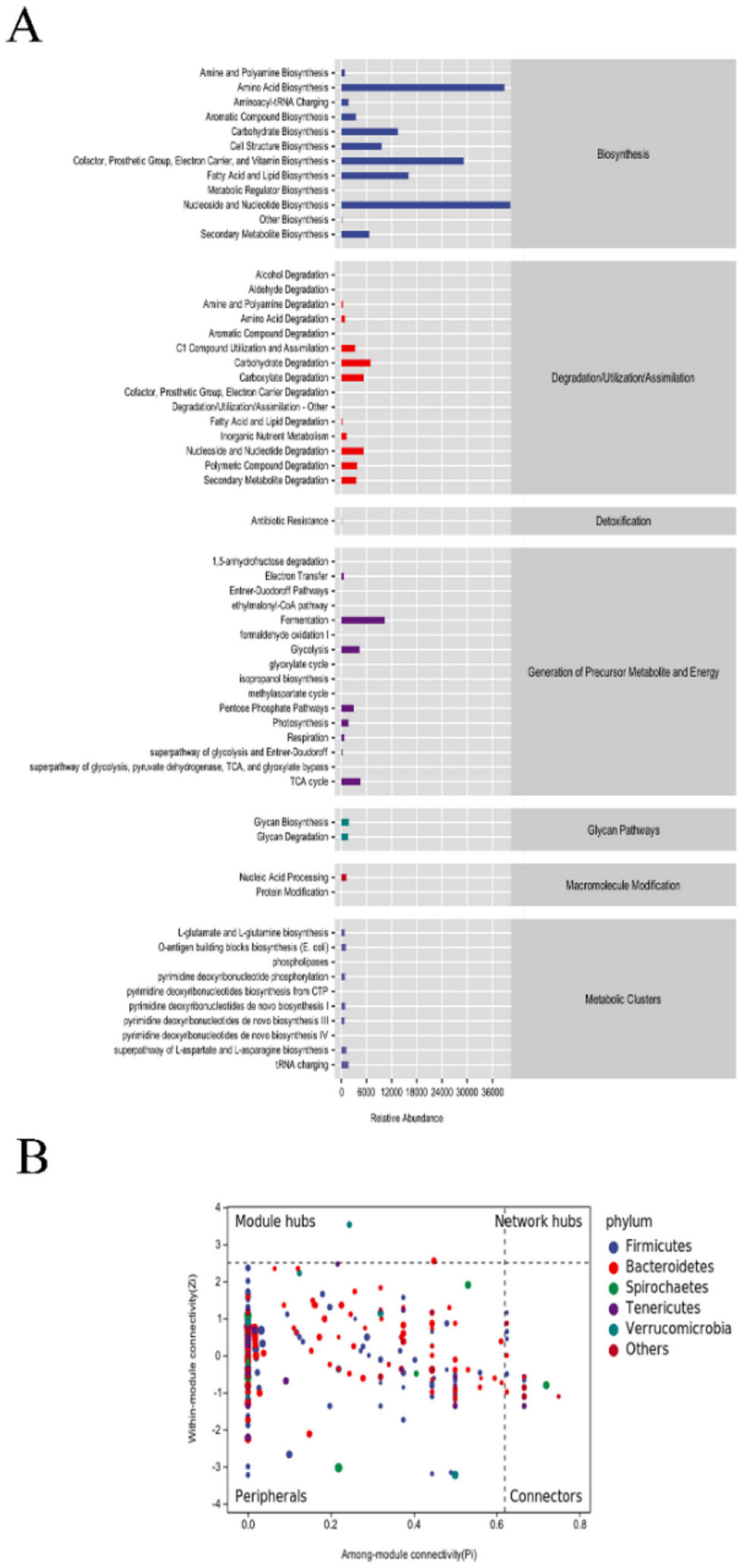
Enrichment of differential microbiota pathways. **(A)** KEGG, MetaCyc, and COG pathways enriched by intergroup differences in microbial communities. **(B)** Phylum-level abundance heatmap.

We performed the KEGG, MetaCyc, and COG analyses on the differential microbiota, and the results are shown in Figure 5A. Differential microorganisms were found to be involved mainly in nucleoside and nucleotide biosynthesis, amino acid biosynthesis, cofactor, prosthetic group, electron carrier, and vitamin biosynthesis, fatty acid and lipid biosynthesis, carbohydrate biosynthesis, carbohydrate degradation, carboxylate degradation, nucleoside and nucleotide degradation, C1 compound utilization and assimilation, polymeric compound degradation, fermentation, glycolysis, the TCA cycle, glycan biosynthesis, and the glycan degradation pathway through pathway enrichment. Additionally, the PIZI graph shows that the five most abundant phyla are Firmicutes, Bacteroidetes, Spirochetes, Tenericulates, and Verrucomimicrobia (Figure 5B).

## Discussion

Adding SB to feed can promote the growth of beef cattle. Moreover, the feed weight ratio decreases significantly after the animals are fed SB, indicating a significant improvement in the feed utilization rate of beef cattle. Adding 5 g/d honeysuckle and 10 g/d SB extract to the lactation diet of sows can significantly increase their feed intake (Liu W. C. et al., 2017). Adding 20–40 g/d BS extract to the diet of cattles can effectively increase their daily feed intake (Lai et al., 2021). Adding 200 mg/kg and 400 mg/kg SB polysaccharides to the diet of broilers can significantly increase their body weight and daily gain (He, 2011). These studies indicate that adding a certain proportion of SB extract to livestock feed can increase feed intake and daily weight gain, decrease the F/G, and improve feed utilization efficiency. The results of this study were consistent with these findings. This may be because the polysaccharides in SB can strengthen the immune system of the animal, increase the number of beneficial bacteria in the intestine, change the permeability of the intestinal wall, and make it easier to absorb and utilize nutrients, thereby improving the growth performance of animals (Xiang et al., 2022).

The results showed that adding SB did not significantly affect the physiological or biochemical indicators of beef cattle. However, after adding SB, the serum TP, GLB, CREA, CHE, MDA, IL-2, IL-6, and TNF-α levels in the G1, G2, and G3 groups were lower than those in the CON group, whereas GLU, IgA, IgG, IgM, SOD, T-AOC, and IL-10 levels were higher. The stems and leaves of SB can decrease the inflammatory response of cattle and strengthen their immune system. This occurred probably because the polysaccharides and total flavonoids in the stems and leaves of SB serve as bioactive immune enhancers, which can strengthen humoral immunity and increase serum immunoglobulin levels. Baicalin can decrease the release of inflammatory factors in the body.

We found that SB and its extracts can significantly affect the structure and diversity of the gut microbiota of animals. Wang et al. reported that SB can regulate the structure and diversity of the gut microbiota in rats with spleen deficiency and damp heat and balance the metabolism of the body (Wang Y. et al., 2022). Fan et al. showed that supplementing plant extracts of SB can regulate oxidative stress and the gut microbiota to alleviate colitis in mice (Wan et al., 2021). However, cattle are susceptible to environmental influences that can cause inflammation and oxidative stress in the body (Putman et al., 2018). Therefore, adding the pure natural Chinese herbal medicine SB may help manage the health of cattle. We added 3%, 6%, and 9% SB to feed to determine the effects of SB additives on the microbial diversity spectrum of cattle feces.

We investigated the effect of adding SB on the microbial diversity profile of cattle feces through 16S RNA sequencing. Our results showed that Firmicutes and Bacteriodota are the two most common phyla in cattle, as reported in other studies (Prasetiyono et al., 2022; Wen et al., 2022; Zhao et al., 2022). We also detected changes in the classification and function of the fecal microbiome among the G1, G2, G3, and CON groups. At the phylum level, Firmicutes and Bacteroidetes were the two main groups, followed by Spirochaetes, Tenericutes, Verrucomimicrobia, and Proteobacteria. This finding matched the results reported by Liu J. et al. (2022) and (Wen et al. 2022). Adding 6% and 9% SB significantly reduced the Firmicutes/Bacteroidetes ratio. Traditionally, the Firmicutes/Bacteroidetes ratio has been associated with disease susceptibility (Ley et al., 2006). Bin reported an increase in the Firmicutes/Bacteroidetes ratio in the jejunum of diarrheal piglets and revealed that an increase in the Firmicutes/Bacteroidetes ratio is related to diarrhea (Bin et al., 2018). The Firmicutes/Bacteroidetes ratio is often used as a marker of obesity in studies (Magne et al., 2020). Members of Firmicutes are associated with obesity because they can ferment plant polysaccharides to produce short-chain fatty acids (SCFAs) to provide additional energy (Ley et al., 2006). *Bacteroides* is an anaerobic gram-negative bacterial genus found in the gut microbiota of mammals (Jandhyala et al., 2015; Rodríguez et al., 2015). Members of Bacteroidetes play an important role in plant polysaccharide decomposition and amino acid fermentation (Dai et al., 2011; Fang et al., 2017). These findings indicate that a decrease in the Firmicutes/Bacteroidetes ratio may weaken the adhesion and colonization of pathogens in the intestine. Therefore, we speculated that adding 3% or 9% SB to feed can improve cattle disease resistance and immunity, reduce diarrhea, and promote healthy growth. (Hua et al. 2021) showed that using Baitouweng Tang (BTWT) to treat ulcerative colitis (UC) in mice increased the relative endurance of Firmicutes, Proteobacteria, Actinobacteria, and Tenericutes. (Nagu et al. 2021) suggested that Bacteroidetes, Firmicutes, Proteobacteria, Teneriicutes, and Verrucomimicrobia regulate neuroinflammation and other inflammatory processes. Therefore, we speculated that adding the stems and leaves of SB to feed may help reduce inflammatory reactions and increase the diversity of the gut microbiota.

At the genus level, *Anaerovibrio, Epulopiscium, Akkermansia, Bacteroides, YRC22, 5-7N15, Butyrivibrio, Prevotella*, and *Anaeroplasma* are markers of intergroup differences. *Anaerovibrio, Epulopiscium*, and *Bacteroides* are significantly correlated with feed efficiency (Bergamaschi et al., 2020). *Anaerovibrio* is also involved in rumen fat degradation (Liu K. et al., 2017). The integrity of the *Akkermansia* intestinal barrier, metabolic health, and immune regulation are closely related. IL-10 is a key anti-inflammatory cytokine that maintains immune homeostasis. The two often exhibit a synergistic relationship in intestinal health and immune regulation (Wang et al., 2024). Zhang et al. and Mu et al. reported that the abundances of *Anaerovibrio, YRC22*, and *Paludibacter* in the rumens of high-yield dairy cattles decreased significantly, indicating that *Anaerovibrio, YRC22*, and *Paludibacter* may be involved in rumen regulation and promote feed absorption to increase milk production in dairy cattles (Zhang et al., 2019; Mu et al., 2019). Huang et al. reported that *5-7N15, Akkermansia*, and *Epulopiscium* were significantly and positively correlated with glycan biosynthesis and metabolism. Catozzi et al. suggested that 5-7N15 is a substance shared by the microorganisms in all healthy milk samples (Catozzi et al., 2017). These findings indicate that adding SB to cattle feed may affect the digestion and absorption of nutrients by the rumen, promote healthy growth of cattle, and even increase milk production. However, further studies are needed to determine the effects of these intergroup differences in microorganisms on the growth and development of cattle.

The functional prediction results showed that the intergroup differential microbiota may participate in amino acid biosynthesis, fatty acid and lipid biosynthesis, carbohydrate biosynthesis, carbohydrate degradation, nucleoside and nucleotide degradation, fermentation, glycolysis, the TCA cycle, glycan biosynthesis, and the glycan degradation pathway. Amino acid metabolism is the central component of protein catabolism. (Song et al. 2022) reported that changes in amino acid metabolism can improve hyperuricemia and inflammation in mice. (He et al. 2018) showed that amino acids can improve the function of the intestinal barrier and the expression of anti-inflammatory cytokines, thereby reducing oxidative stress, apoptosis of intestinal cells, and the expression of proinflammatory cytokines during intestinal inflammation. Amino acid metabolism plays an important role in preventing and treating intestinal inflammation. Strengthening the amino acid metabolism pathway may alleviate the inflammatory response in cattle. The TCA cycle is closely associated with amino acid metabolism, glucose metabolism, and fat metabolism. This finding is consistent with the findings of our study, as we found that the intergroup differential microbiota is involved in fatty acid and lipid biosynthesis, carbohydrate biosynthesis, glycolysis, and the TCA cycle. Lipids can regulate various cellular responses, including cell growth and death, as well as inflammation, through receptor-mediated pathways (Bennett and Gilroy, 2016). Macrophages rely mainly on aerobic glycolysis and fatty acid biosynthesis to maintain the inflammatory response and kill pathogens. Glycolysis is a rapid method for ATP production (Chandel, 2021), and fatty acids are precursors for the synthesis of inflammatory mediators (Innes and Calder, 2018). Macrophages can metabolize fatty acid oxidation and oxidative phosphorylation to mediate the regression of inflammation (Bennett and Gilroy, 2016). The TCA cycle is involved in the activation of inflammatory macrophages (Lampropoulou et al., 2016). Kelly et al. reported that the TCA cycle intermediate succinate promotes the expression of inflammatory genes (Kelly and O'Neill, 2015). Han et al. reported that by inoculating degrading microorganisms with high hydrolytic ability, the glycolytic metabolic pathway can be promoted, digestion can be significantly accelerated, and the generation of metabolic energy can be increased (Han et al., 2024). Chen et al. reported that adding *Litsea cubeba* essential oil to feed can significantly improve growth performance, blood antioxidant capacity, immune function, and digestibility of nutrients in pigs. This may be caused by the change in the structure of the fecal microbiota due to *L. cubeba* essential oil (Chen F. et al., 2023). To summarize, our findings indicated that adding SB may affect metabolic pathways such as amino acid metabolism, carbohydrate and lipid synthesis, glycolysis, and the TCA cycle by improving the intestinal microbial structure, thereby reducing the inflammatory response in cattle, regulating the oxidative stress state, and promoting the healthy growth of cattle.

We analyzed the production performance and serum biochemical indices of cattle, as well as the metagenome of their feces. However, further studies are needed to comprehensively elucidate the mechanisms by which the replacement of corn silage with SB can improve weight gain and anti-inflammatory and antioxidant effects in beef cattle. The physiological status of cattle (such as age, health condition, diet, etc.) and the environmental factors while breeding (such as temperature, humidity, stocking density, etc.) affect their intestinal microbiota. The changes in these factors may lead to dynamic changes in the structure and function of the microbiota, making it difficult for the microbial sequencing results to accurately reflect the true relationship between the microbiota and the health and production performance of cattle. Therefore, multiomics analyses, such as metabolomics, genomics, and transcriptomics, are needed to elucidate the mechanisms by which replacing corn silage with SB improves weight gain and anti-inflammatory and antioxidant effects in beef cattle to provide new feeding materials and strategies for breeding local beef cattle in China. Meanwhile, due to the small sample size of our experiment (n=5 per group), this may affect the statistical power and generalizability of the research results. In the future, larger scale and longer-term animal feeding experiments are needed to determine the long-term impact and economic feasibility of SB as a substitute.

## Conclusion

To summarize, adding different proportions of SB alters the structure of the intestinal microbiota of cattle. The structural changes in the microbiota associated with these differences are closely associated with the inflammatory response and the digestion and utilization of nutrients in the rumen. In this study, we found that the microbial community changed after SB was added, which may have beneficial characteristics. However, larger-scale and longer-term animal feeding experiments are needed to determine the long-term effects and economic feasibility of SB as a substitute.

## Data Availability

The original contributions presented in the study are publicly available. This data can be found here: https://www.ncbi.nlm.nih.gov/, accession number PRJNA1096382.
